# Combining ultrasonography and noncontrast helical computerized tomography to evaluate Holmium laser lithotripsy

**DOI:** 10.1097/MD.0000000000005564

**Published:** 2016-12-09

**Authors:** Jia Mi, Jie Li, Qinglu Zhang, Xing Wang, Hongyu Liu, Yanlu Cao, Xiaoyan Liu, Xiao Sun, Mengmeng Shang, Qing Liu

**Affiliations:** a Qilu Hospital of Shandong University; b Department of Ultrasound, Shandong Traffic Hospital; c Department of Ultrasound, Qilu Hospital of Shandong University, Jinan, Shandong, China; d Department of Microbiology and Immunology, East Carolina University Brody School of Medicine, Greenville, NC; e Department of Pulmonology, Peking Union Medical College Hospital, Peking Union Medical College, Chinese Academy of Medical Sciences, Beijing, China.

**Keywords:** calculus, holmium laser lithotripsy, noncontrast helical computerized tomography, ultrasonography

## Abstract

The purpose of the study was to establish a mathematical model for correlating the combination of ultrasonography and noncontrast helical computerized tomography (NCHCT) with the total energy of Holmium laser lithotripsy.

In this study, from March 2013 to February 2014, 180 patients with single urinary calculus were examined using ultrasonography and NCHCT before Holmium laser lithotripsy. The calculus location and size, acoustic shadowing (AS) level, twinkling artifact intensity (TAI), and CT value were all documented. The total energy of lithotripsy (TEL) and the calculus composition were also recorded postoperatively. Data were analyzed using Spearman's rank correlation coefficient, with the SPSS 17.0 software package. Multiple linear regression was also used for further statistical analysis.

A significant difference in the TEL was observed between renal calculi and ureteral calculi (*r* = –0.565, *P* < 0.001), and there was a strong correlation between the calculus size and the TEL (*r* = 0.675, *P* < 0.001). The difference in the TEL between the calculi with and without AS was highly significant (*r* = 0.325, *P* < 0.001). The CT value of the calculi was significantly correlated with the TEL (*r* = 0.386, *P* < 0.001). A correlation between the TAI and TEL was also observed (*r* = 0.391, *P* < 0.001). Multiple linear regression analysis revealed that the location, size, and TAI of the calculi were related to the TEL, and the location and size were statistically significant predictors (adjusted *r*^2^ = 0.498, *P* < 0.001).

A mathematical model correlating the combination of ultrasonography and NCHCT with TEL was established; this model may provide a foundation to guide the use of energy in Holmium laser lithotripsy. The TEL can be estimated by the location, size, and TAI of the calculus.

## 1 Introduction

After being investigated and demonstrated to be safe and effective in 1960, a variety of laser techniques have been used in urological procedures. Because of its precision and strong decomposing power, the Holmium laser has become one of the most popular tools in urological procedures, including lithotripsy. One major advantage of the Holmium laser is that it can fragment urinary calculi efficiently, regardless of size, hardness, chemical composition, and physical consistency; accordingly, a high stone-free rate can be achieved.^[1]^ Another advantage of the Holmium laser is that it has a wavelength of 2100 nm, which is nearly completely absorbed by water, and it has a tissue penetration of <0.5 mm, making it safe to ablate and coagulate tissue, with minimal damage to surrounding areas.^[2]^ Thus, the Holmium laser has become the primary choice to fragment calculi.

The mechanism of Holmium laser lithotripsy has been investigated for many years. Many studies have suggested that it might result from the photoacoustic effect generated by pulsed lasers. When pulsed lasers are fired, cavitation bubbles are produced at the water–calculus interface. Shock waves are produced by continuous rebound, and the bursting of the bubbles is transmitted to the calculus and can cause calculus fragmentation.^[3]^ However, Dushinski and Lingeman^[4]^ have proposed that the mechanism of fragmentation should be explained by the photothermal effect on urinary calculi. Chan et al^[5]^ have also reported that the Holmium laser lithotripsy mechanism is primarily a photothermal effect through which the Holmium laser increases the temperature of the irradiated area, causing a chemical breakdown of the calculi and weakening the physical strength of the irradiated area, whereas interstitial water and vapor expansion merely facilitate the drainage of the fragments within the area.

Several parameters that affect the fragmentation efficiency of Holmium laser lithotripsy have been assessed, including the pulse energy, pulse duration, frequency, and power setting. Sea et al^[6]^ have reported that the fragmentation rate does not increase when the frequency increases and that the pulse energy is constant. Chawla et al^[7]^ have shown that the fragmentation rate increases with the pulse energy, but it does not consistently increase with the pulse frequency. It has been reported that in cases of high density and fixed calculi, a higher pulse energy, and shorter pulse duration will lead to increased fragmentation efficacy.^[8]^ In an in vitro study, Peter and Olivier^[9]^ observed that a low frequency-high pulse energy setting is more efficient than a high frequency-low pulse energy setting at the same power levels; they also found linear correlations between the pulse energy and fragmentation size, as well as the fissure width and the depth.

Different energy is needed to fragment calculi with different characteristics. Unguided use of energy in laser lithotripsy has some disadvantages. Insufficient energy cannot fragment calculi effectively, whereas overused energy may lead to a higher incidence rate of complications and overconsumption. Preoperative evaluation of the total energy of Holmium laser lithotripsy can predict the operation difficulty, estimate the operation time, and make emergency program to high-risk patient in advance. Therefore, guiding the use of energy plays a key role in Holmium laser lithotripsy, and it may improve the safety and efficiency of laser lithotripsy and increase the cost-effectiveness of its operation.

First introduced in the early 1990s, the mechanism of Holmium laser lithotripsy and the parameters for fragmentation efficiency have been frequently investigated. However, no in vivo studies have reported a correlation between the combination of ultrasonography and noncontrast helical computerized tomography (NCHCT) and the total energy of lithotripsy (TEL), particularly the potential role of ultrasonography in the assessment of TEL, such as twinkling artifact and acoustic shadowing (AS). The purpose of this study was to establish a mathematical model to correlate the combination of ultrasonography and NCHCT with the total energy of Holmium laser lithotripsy.

## 2 Materials and methods

### 2.1 Patients

This study proposal was approved by the institutional review board. A total of 180 patients with a single renal or ureteral calculus were enrolled in the study from March 2013 to February 2014. All patients provided written informed consent prior to study enrollment.

### 2.2 Ultrasonography

**Figure 1:**
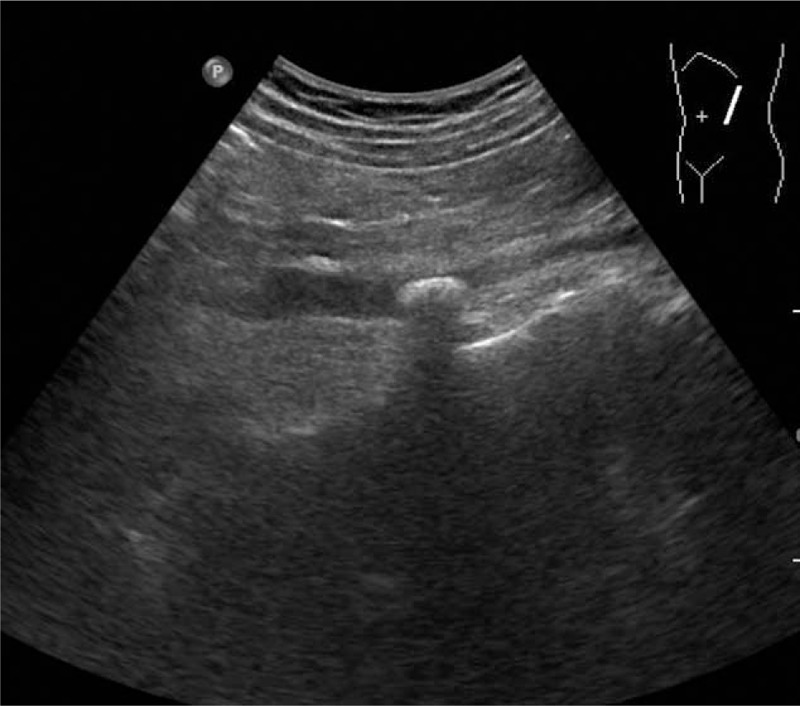
Gray-scale imaging to measure the calculus size and to confirm the calculus location and AS level. AS = acoustic shadowing.

**Figure 2:**
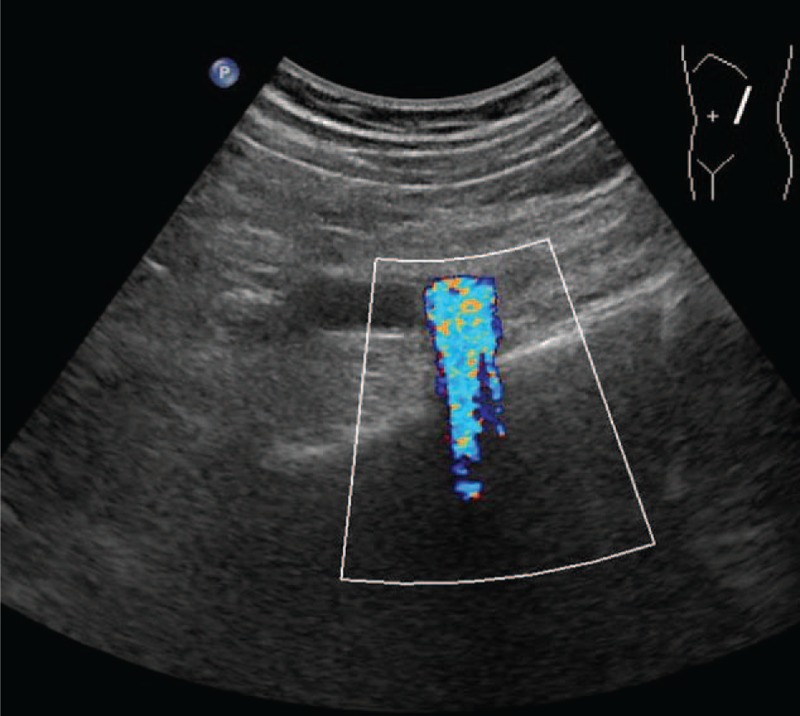
Color Doppler imaging to assess twinkling artifacts.

All examinations were performed by a single ultrasonologist (with 7 years of experience) using a Philips iU22 ultrasound scanner (Philips Medical Systems, Bothell, WA) using a C5–1 curvilinear array probe. All patients underwent ultrasonography the day before lithotripsy. No special preparation was required for the patients with kidney or proximal/middle ureter calculi. Patients with calculi in the distal ureter were requested to moderately fill their bladders. Scanning started with gray-scale imaging to measure the calculus size and to confirm the calculus location and the presence or absence of AS (Fig. 1). After the largest and clearest image of the calculus was obtained, the patients were requested to hold their breath, and a cine loop of the calculus was recorded for 10 seconds. Color Doppler imaging was used to assess twinkling artifacts (Fig. 2). When the maximum and steadiest twinkling was detected, the patients were requested to hold their breath again, and another 10-second cine loop of the twinkling artifact was recorded. All cine loops were saved for further analysis.

Philips DICOM Viewer R2.5L1-SP3 software was used for data processing. Five static images were randomly chosen from each cine loop replay for the statistical analysis. In gray-scale imaging, the calculus location was confirmed and classified as renal or ureteral. The maximum calculus size was measured and classified into 4 grades: smaller than 10 mm was grade 1; between 10 and 20 mm was grade 2; between 20 and 30 mm was grade 3; and larger than 30 mm was grade 4. AS was assessed and classified into 2 levels: calculus without AS was level 0 and calculus with AS was level 1. There are no uniform or strict criteria to evaluate the intensity of twinkling artifacts, which makes the evaluation subjective and operator-dependent. Referring to Gao et al^[10]^ and Wang et al,^[11]^ we quantified the twinkling artifact intensity (TAI) by calculating the number of color pixels in each artifact. In Adobe Photoshop CS5, the background gray pixels were deleted, the background was masked in black, and the images containing only color pixels from twinkling were saved. Image J, an image editing application for medical image processing, was then used to select a region of interest that included all color pixels in the artifact and some background pixels and to draw a histogram of the region. The number of color pixels in the artifact was calculated by subtracting the number of background pixels from the number of all pixels in the region. All data were obtained from measuring 5 different images, and the average of the 5 measurements was used as the final result.

### 2.3 Computed tomography

Immediately after undergoing ultrasonography, all patients were examined using noncontrast helical computerized tomography (NCHCT) with a Philips Brilliance iCT 256 scanner (Philips Healthcare, Cleveland, OH). The CT value of each calculus was generated, as follows: Hounsfield units were measured for 3 different 0.02 cm^2^ regions of interest on each calculus. The average of the 3 measurements was used as the final CT value for each specific calculus. The CT values of the calculi were classified into 4 grades: below 400 Hu was grade 1, between 400 and 800 Hu was grade 2, between 800 and 1200 Hu was grade 3, and over 1200 Hu was grade 4.

### 2.4 Lithotripsy

All lithotripsies were performed by 2 surgical urologists (1 with 5 years of experience and the other with 7 years of experience) using a dual-wavelength Holmium laser therapeutic machine (Power Suite 80/100w, Israel). The following laser settings were used: 200 μm laser fiber at output energy of 0.5/0.6 J and a pulse repetition rate of 20/35 Hz. During lithotripsy, fragments were targeted to obtain the smallest size possible. Using the laser fiber as a frame of reference, all fragments >4 mm were removed with a basket catheter. The total energy of each completed laser lithotripsy was recorded. The criterion for a completed laser lithotripsy was no residual fragment left, or, if any, the fragment size had to be ≤4 mm, and spontaneous excretion of the fragment was expected. The efficacy of lithotripsy was evaluated 1 to 2 days after the procedure through ultrasonography and plain film of the kidney–ureter–bladder.

### 2.5 Calculus composition analysis

After the completion of each laser lithotripsy, 1 fragment was randomly chosen for composition analysis through automatic infrared spectrophotometry with the Medical Automated FTIR Human Calculi Analysis System (LIIR-20, Lanmode Scientific Instrument Co. Ltd).

### 2.6 Statistical analysis

Statistical analyses were performed with the SPSS 17.0 software package. Spearman's rank correlation analysis was used to assess the correlations between the TEL and the calculus location, size, AS level, TAI, and CT value, as well as the calculus composition. Multiple linear regression was performed to formulate a mathematical model to estimate the TEL. A *P*-value of < 0.05 was considered to be statistically significant.

## 3 Results

Of the 180 patients who participated in the study from March 2013 to February 2014, 5 patients were excluded from the final analyses because their lithotripsies were evaluated as failed: in 2 cases, the renal calculi in the subrenal calyx were too secluded to be reached and fragmented effectively, and 3 patients had residual fragments >4 mm after the procedure. Therefore, valid data for this study were collected from 175 patients (115 females and 60 males; average age, 48.6 ± 13.0 years; age range, 23–84 years). Fifty-one patients had renal calculi, and 124 patients had ureteral calculi. The calculi had an average size of 14.7 ± 6.1 mm (6–41 mm), and 46 of them were grade 1, 101 were grade 2, 22 were grade 3, and 6 were grade 4. In terms of AS, 16 calculi were level 0, and 159 were level 1. In terms of CT values, 7 calculi were grade 1, 47 were grade 2, 72 were grade 3, and 49 were grade 4 (Table 1). For the calculus composition analysis, 66 calculi had a single component, including 52 calcium oxalate monohydrate calculi and 14 uric acid calculi, and 109 calculi were a mixture of 2 to 4 types of components.

**Table 1:**
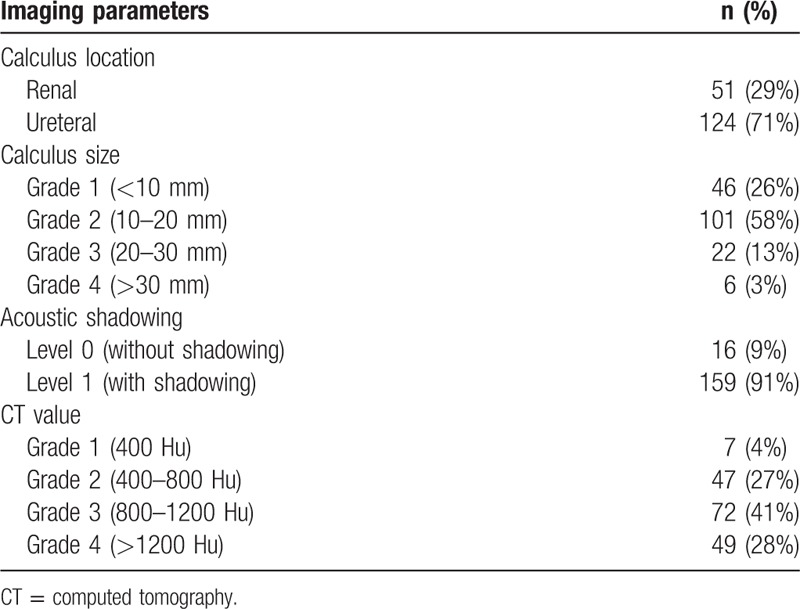
Imaging parameters of the calculi.

There was a significant difference between the TEL required for renal calculi and that required for ureteral calculi (*r* = –0.565, *P* < 0.001), and the TEL for renal calculi was much higher than that required for ureteral calculi. Statistical analysis showed a strong correlation between the size of the calculus and the TEL (*r* = 0.675, *P* < 0.001), and larger calculi required a higher TEL. There was also a significant difference in the TEL between different levels of AS (*r* = 0.325, *P* < 0.001), and calculi with AS required more energy for fragmentation. TAI was strongly correlated with TEL (*r* = 0.391, *P* < 0.001), and a higher TEL was required for calculi with a greater TAI. A correlation between the CT value of the calculus and the TEL was also observed (*r* = 0.386, *P* < 0.001), and the energy required for lithotripsy was proportional to the CT value of the calculus. There were no statistically significant differences between the TEL values for the different calculi compositions (*r* = –0.133, *P*>0.05, Table 2).

**Table 2:**
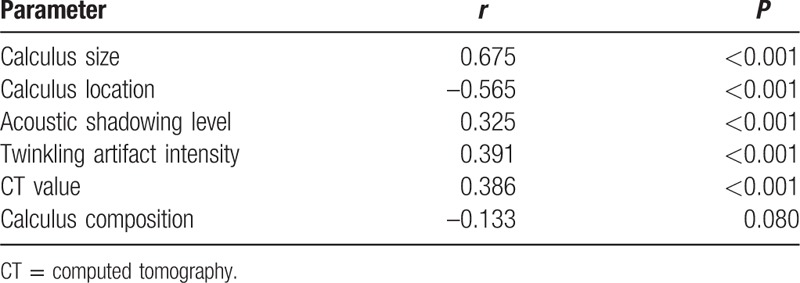
Results of Spearman's rank correlation analysis of the imaging parameters and total energy of lithotripsy.

**Figure 3:**
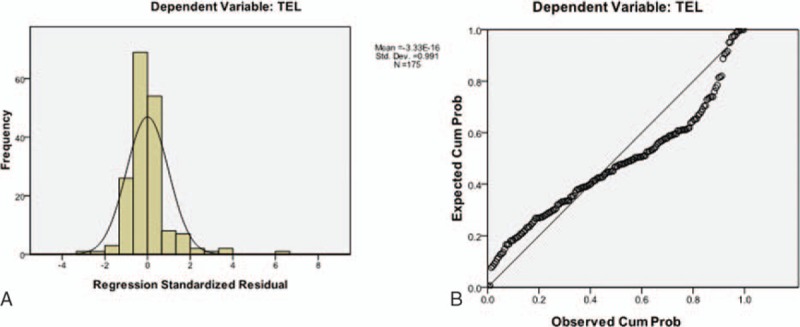
A normality test showed that the standardized residual aligned with the normal distribution, which met the applied condition of the multiple linear regression model. TEL = total energy of lithotripsy.

To further eliminate confounding factors and to estimate the TEL in patients with different characteristics, a multiple linear regression model was built with the following parameters: calculus location (1 = renal calculus and 2 = ureteral calculus), AS level (0 = calculus without AS, 1 = calculus with AS), size of calculus, TAI and CT value (Table 3). After collinearity was eliminated and normality was tested (Fig. 3), the following multiple linear regression equation was obtained: TEL (J) = 755.4 × size of calculus (mm)–3099.4 × location of calculus (1 = renal calculus; 2 = ureteral calculus)+0.2 × TAI (*F* = 58.644, adjusted *r*^2^ = 0.498, *P* < 0.001). The equation shows that more energy is needed for lithotripsy in patients with larger calculi, renal calculi, or calculi with greater TAI.

**Table 3:**
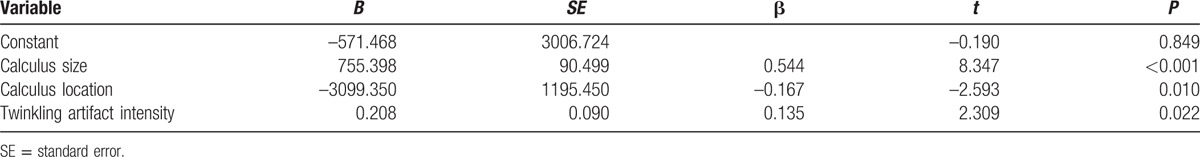
Results of multiple linear regression analysis of the variables estimating total energy of lithotripsy (n = 175).

## 4 Discussion

A mathematical model correlating the combination of ultrasonography and NCHCT with TEL was established in our study, and it may provide a foundation to guide the use of energy in Holmium laser lithotripsy. The TEL can be estimated by the location, size, and TAI of the calculus. By providing an initial estimate of the total energy required in Holmium laser lithotripsy, the combination of ultrasonography and NCHCT is likely to improve the safety and efficiency of laser lithotripsy, as well as the cost-effectiveness of the procedure, by avoiding overconsumption of pulse energy during the operation.

In a previous retrospective study, Molina et al^[12]^ analyzed the differences in cumulative Holmium laser energy in different locations. They concluded that renal calculi required more energy than ureteral calculi. We reached a similar conclusion: the required TEL differed significantly between the kidney and the ureter. Although the exact mechanism is still not clear, 1 conceivable explanation for the phenomenon is that hydronephrosis or hydrocalycosis around the renal calculus makes it more mobilizable than ureteral calculi, and the total amount of contact between the calculus and the tip of the laser fiber is decreased. Therefore, more pulses are likely to be fired inefficiently, and more energy may be wasted during the fragmentation of renal calculi.

The characteristics of calculi play an important part in the laser lithotripsy procedure. In terms of size, a larger calculus requires more energy for fragmentation. Blomley et al^[13]^ have performed a systematic review of Holmium laser use for calculus lithotripsy. They reported that the required cumulative energy of pulses increased with the calculus size and the mass. This finding is consistent with our finding that the correlation between the calculus size and the TEL is statistically significant.

Note that for the first time, our study demonstrated a significant difference between the TEL at different AS levels. This phenomenon has 2 explanations. First, acoustic impedance plays an important role in the intensity of AS. A larger difference in the acoustic impedance between materials will lead to a stronger reflection at the interface and a greater AS. The higher the calculus density, the larger its acoustic impedance and the greater its AS. Second, a higher density calculus requires more energy from laser lithotripsy for fragmentation. After investigating the characteristics of BegoStone, Liu and Zhong^[14]^ observed that it was much denser and harder than plaster of Paris, and it was more difficult to fragment in SWL compared to plaster of Paris. In an in vitro study, Wezel et al^[8]^ reported that at all tested settings, the fragmentation efficiency was remarkably higher for soft calculi than for hard calculi. Kronenberg and Traxer^[15]^ compared the fragmentation efficiency among artificial calculi constructed from different materials. They found that the ablation rate was higher in calculi made of plaster of Paris than those made from BegoStone Plus. They also concluded that a hard calculus material was more difficult to ablate than a soft calculus material at the same laser lithotripter settings. According to these studies, it is reasonable to conclude that the energy required for calculus fragmentation through laser lithotripsy is proportional to the density of the calculus.

We also noticed a significant correlation between TAI and TEL that has not been previously reported. However, due to many potential influential factors from the twinkling artifact, we could not clearly define the correlation. One possible explanation is the correlation between the calculus size and TAI. In an in vitro study, Louvet^[16]^ found a significant correlation between the twinkling artifact grade and the calculus size and deduced that a higher artifact grade should be expected for larger calculi. Considering that both TAI and TEL are correlated with calculus size, a correlation between TAI and TEL should also be reasonably expected.

In alignment with previous studies, a significant correlation between TEL and CT values of the calculus was also observed in our study. In a retrospective study, Ito et al^[17]^ divided patients into low and high attenuation coefficient groups based on the preoperative median absolute CT values for average calculus density, and they found the fragmentation efficiency was significantly higher in the low attenuation coefficient group compared to the high group. Molina et al^[12]^ have also reported that calculus size and hardness (measured by NCHCT) were important predictors for cumulative laser energy.

The role that calculus composition plays in laser lithotripsy efficiency remains less defined. In an in vitro study, Teichman et al^[18]^ observed that when the laser fiber and the energy level remained unchanged, the fragmentation efficiency of the Holmium laser varied among different calculus compositions. They attributed this phenomenon to the different temperature thresholds of different compositions. Molina et al^[12]^ reported that all calcium calculi require less energy compared with uric acid calculi in a univariate analysis. However, we failed to find a statistically significant difference in the TEL among different compositions of calculi. Although the reason for this observation is not clear, the possible explanations are as follows. First, only 1 fragment was collected per calculus for the composition analysis, which might not accurately represent the calculus as a whole. Second, although we could determine the composition of a calculus, we were unable to define the proportion of each component of the calculus because of the functional limit of the calculi analysis system that we used. Therefore, the statistical outcome of the correlation between the TEL and the calculus composition could be affected.

This study has several limitations. First, a patient's BMI might affect the TAI because of the change of depth to the calculus, which we failed to consider in our study. Second, we could not pre-set and optimize the machine settings for evaluating the TAI more sensitively and specifically. In addition, we performed the study using equipment from only 1 manufacturer, and it is possible that different results could be obtained using different machines. The factors above may have caused deviations; thus, further investigation is needed.

## 5 Conclusion

A mathematical model correlating the combination of ultrasonography and NCHCT with TEL was established; this model may provide a foundation to guide the use of energy in Holmium laser lithotripsy. The TEL can be estimated by the location, size, and TAI of the calculus.

## Acknowledgments

This manuscript was edited for English language by American Journal Experts (AJE). The authors are particularly grateful to Yuanyuan Liu for her assistance with the statistical analysis. They also thank Mingjie Li and Xiangtao Wang for their technical advice about the Holmium laser lithotripsy.
